# The initial development and validation of a child-oriented food literacy questionnaire

**DOI:** 10.1017/S1368980025101651

**Published:** 2025-12-29

**Authors:** Ilse van Lier, Britt van Belkom, Edgar van Mil, Remco Havermans

**Affiliations:** 1 Chair Group Youth, Food & Health, Maastricht University Campus Venlo, Venlo, the Netherlands; 2 Department of Paediatrics, Children’s Lifestyle Medicine Centre, Jeroen Bosch Hospital, ‘s-Hertogenbosch, the Netherlands; 3 Laboratory of Behavioural Gastronomy, Maastricht University Campus Venlo, Venlo, the Netherlands

**Keywords:** Food literacy, Children, Instrument development, Farm-to-fork, Questionnaire

## Abstract

**Objective:**

The Dutch Children’s Food Literacy Questionnaire (DCFLQ) was developed and validated to assess food literacy among children aged 8–12 years. The DCFLQ is structured around farm-to-fork principles, including questions on food production, distribution, consumption, waste, and sustainability.

**Design:**

After initial item pool creation, the DCFLQ was developed in collaboration with experts and children. The validation process included assessments of reliability and construct validity, as well as a test–retest evaluation in a subgroup of children.

**Setting:**

The expert panel consisted of domain-related researchers, a pedagogue, a paediatrician, dietitians and a primary school teacher. Children were recruited via primary schools and a sports club.

**Participants:**

A total of eleven experts and twenty-seven children participated in the development process; 608 children participated in the validation process.

**Results:**

The final questionnaire comprised twenty-nine questions and demonstrated good internal consistency (Cronbach’s *α* = 0·80) and test–retest reliability (ICC = 0·81). DCFLQ scores positively correlated with age, indicating that food literacy is higher in older children.

**Conclusions:**

The DCFLQ is a valuable tool for assessing the effectiveness of nutrition intervention programmes and monitoring Dutch children’s food literacy over time. International expert consensus on developing food literacy instruments is needed, as diversity in assessment tools impedes cross-cultural comparisons.

The rise in overweight and obesity levels worldwide is a worrying trend^(1)^; it is expected that in 2025 nearly one out of four children lives with overweight or obesity^(2)^. Childhood obesity has significant immediate health implications. Childhood overweight often persists into adulthood^(3)^, and obesity at a young age increases the risk of developing non-communicable diseases later in life^(4,5)^. Furthermore, being obese may affect children’s self-esteem and can cause physiological issues^(6–8)^.

The increase of unhealthy dietary habits can partly be attributed to changes in food systems^(1)^. Modern food environments are characterised by the widespread availability, affordability, and marketing of energy-dense and nutrient-poor foods^(9)^. In addition, the time spent on preparing food has decreased, resulting in an increased dependence on convenience foods. This shift towards spending less time on cooking also impacts the transmission of food-related knowledge and skills from parents to children, whereas numerous studies suggest that involvement in meal preparation at home facilitates healthier dietary intake^(10–12)^.

To ensure that children can navigate in a world that promotes nutrient-poor and energy-dense foods and beverages, it is essential to educate them about food and nutrition from a young age onwards^(13)^: in other words, making them food literate.

Food literacy captures the knowledge, behaviours and skills needed to make informed and deliberate food-related choices. It includes the entire food process from seed to plate and incorporates aspects such as social norms, sustainability and culture^(14–16)^. However, consensus on the definition of food literacy has not yet been reached^(17)^. Some researchers have defined food literacy in the light of health literacy, in which three levels can be distinguished: a functional level (capturing knowledge), an interactive level (focused on the translation of knowledge into practice and the development of skills) and a critical level (acting on and influencing barriers to good nutrition)^(18)^. Although this model provides a useful structure for understanding the multidimensional nature of food literacy, young children may not yet have the cognitive maturity to engage in actions that challenge structural barriers to healthy food environments. One of the most commonly cited definitions of food literacy comes from Vidgen and Gallegos^(16)^. They define food literacy as the knowledge, skills and behaviours needed to plan, manage, select, prepare and eat food to meet needs and determine intake, thereby enhancing dietary resilience over time. Also here, its applicability to younger children may be limited, as the cognitive and practical demands of planning and managing food intake may exceed their developmental capacities.

Among adults and adolescents, several tools have been developed to measure food literacy^(19,20)^. Positive associations have been found between food literacy, diet quality and food behaviour in both adults^(21–23)^ and adolescents^(24,25)^. For example, adolescents with proficient cooking *skills* tend to have healthier eating patterns, such as greater consumption of fruits and vegetables, reduced fast food intake and more frequent home-cooked meals^(25)^, and both adolescents and adults with greater food *knowledge* tend to have healthier dietary practices^(23,24)^. Food literacy can reduce reliance on convenience foods by increasing competence in food preparation, but it may also lead to greater awareness of nutrition recommendations, which in turn may support more deliberate food choices^(22,26)^. However, social and environmental factors, such as peer influence and food access, can moderate or mediate the influence of food literacy on eating behaviour. For instance, an adolescent with high food literacy may still maintain unhealthy dietary practices when their friends do so^(27–29)^. Although research on food literacy in adults and adolescents is well established, studies on children’s food literacy are scarce^(30)^. Few studies have focused on the development of food literacy assessment tools for children^(31–33)^. Cultural food diversity further complicates the development of food literacy tools, as foods, dietary habits, meal preparation techniques and traditions may vary widely across different cultures. Therefore, food literacy tools cannot readily be applied across diverse culinary cultures. A food literacy tool for Dutch consumers would refer to different foods, meals, cooking skills and behaviours than does a, for example, Brazilian or Indonesian food literacy assessment. Also, as children’s cognitive ability is still developing and they largely rely on their parents when it comes to nutrition and food, not all components of food literacy as identified by Vidgen and Gallegos are relevant for children. For instance, children under the age of 12 years typically lack the autonomy to independently plan and manage their food patterns.

For children, food literacy might better be approached through a farm-to-fork (F2F) principle as it is a tangible way to help children understand where food comes from, how it is produced and what impact it has on their health and the environment^(34)^. The F2F principle encompasses all components of the food system, from agricultural production (farm) to consumption (fork), and includes several components such as food production, processing, distribution and consumption. The F2F principle also integrates sustainability (i.e. the environmental impact, including organic production and food waste) and cultural aspects of food systems. In the current study, this principle is used as a basis for the development and validation of a food literacy assessment tool for children: the Dutch Children’s Food Literacy Questionnaire (DCFLQ). The questionnaire is designed for children aged 8–12 years, a developmental stage characterised by an increased awareness of the world, including the food system, and the development of skills such as preparing simple meals^(15,35)^. This underscores the importance and relevance of becoming food literate during this period. The current study describes the development and validation of a food literacy questionnaire for children in the Netherlands, which can ultimately be used to measure their food literacy status.

## Methods

### Study design

**Figure 1.**
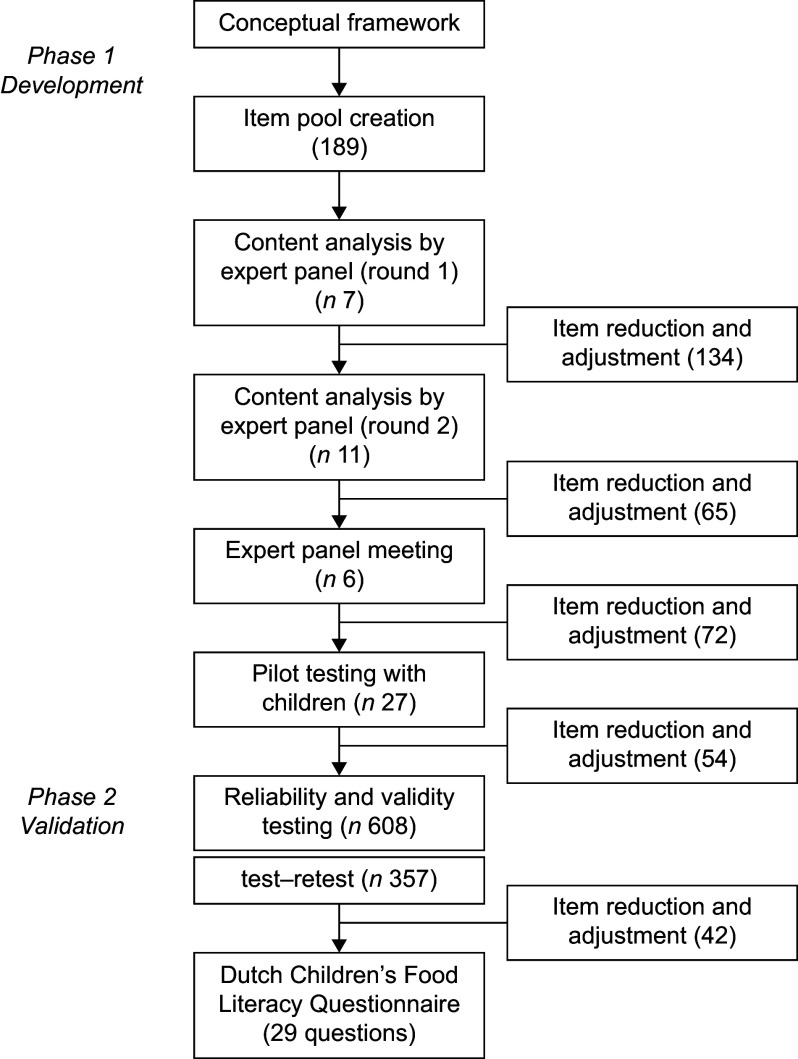
Overview of the study design for the development and validation of the Dutch Children’s Food Literacy Questionnaire (DCFLQ). The bold numbers between brackets refer to the number of items that were left after item reduction and adjustment. The final DCFLQ comprises twenty-nine questions containing a total of forty-two items.

The current study employed a mixed-methods approach and was divided into two phases (see Figure 1). Phase 1 focused on the development of the DCFLQ, including item pool creation, feedback from experts and pilot testing with children. In the second phase, the questionnaire was validated through large-scale testing with a group of children. Recommendations for instrument development, such as those described by Streiner and Kottner (2014)^(36)^, were used as general guidance.

### Phase 1: Questionnaire development

#### Conceptual framework

In the current research, we focused on the ‘farm-to-fork’ principle as a base for food literacy. This principle is categorised into the following domains: food production, food processing and distribution, food consumption, and food loss and food waste, with sustainability integrated in each of the four domains. After item pool creation (see below), we further divided these domains into subdomains to provide a structured and comprehensive framework for food literacy; see Table 1. The subdomains were developed through an iterative process in which we critically reviewed the thematic content of the domains and the draft items and grouped items that reflected similar underlying concepts. This preliminary structure was subsequently evaluated by experts (*n* 7) in food literacy, child nutrition and education, whose feedback informed the final subdomain categorisation. Although certain constructs may span multiple domains, each subdomain was assigned to the domain with the strongest conceptual alignment.

**Table 1.** Food literacy with farm-to-fork principles^(34)^ as domains and with corresponding subdomains, including descriptions per subdomain

Farm-to-fork principles	Subdomains	Description	Final # items
Food production	Origin (2)	Locally and globally produced foods	1
	Source (7)	Animal- and plant-based foods	3
	Growing process (8)	Production of raw foods	4
Food processing and distribution	Food processing (4)	Processing food, including harvesting and packaging	2
	Food distribution (2)	Shipping methods	0
	Food quality (2)	Quality of the food processed	0
Food consumption	Ingredient knowledge (21)	Knowledge of different ingredients used in cooking	12
	Nutrition and health (14)	Knowledge of the effects of nutrition on health; knowledge of the health components of ingredients	4
	Food labels and nutrition information (12)	Knowing where to find product information and the ability to correctly interpret the information	3
	Diet composition (9)	Knowledge of meal components and what items to select to have a balanced diet	4
	Cooking skills (6)	Ability to safely use cooking utensils	1
	Food safety (11)	Good hygiene practices	2
	Social norms (4)	Interpreting eating norms	1
	Culture and traditions (10)	Being aware of food of other cultures and corresponding traditions	1
	Food self-efficacy (5)	The belief in one’s ability to make food-related choices	2
Food loss and waste prevention	Reducing food waste (12)	Knowing how to avoid food waste, taking care of shelf life	1
	Recycling food (2)	Being able to correctly recycle food and food waste	1
	Environmental impact (3)	Being aware of the environmental impact of food waste	0

Note that sustainability is not treated as a separate subdomain; instead, questions related to sustainability are integrated across the various subdomains. The numbers in parentheses () indicate the number of questions included in the initial development of the DCFLQ, based on the questions derived after the first expert round.

#### Item pool creation

Items were drawn from existing (child-oriented) food literacy tools and general nutrition education questionnaires^(15,33,37–44)^. These items were categorised into one of the four domains of food literacy and were later divided into subdomains based on their thematic similarity (see Table 1). A comprehensive list of 189 items was derived from these existing questionnaires. These items were translated into Dutch and were formatted into statements with three answering options: ‘true’, ‘false’ or ‘don’t know’.

#### Expert panel

The item list was presented three times to a group of experts from different disciplines including a dietitian, a pedagogue, a primary school teacher, a paediatrician, a psychologist and domain-specific experts. Panellists were recruited through the research team’s network and approached via email. They were asked to critically evaluate the relevance of the items. In total, fifteen experts were invited, of whom eleven agreed to participate in at least one round of the panel.

#### Pilot testing with children

The comprehensibility and clarity of the questions obtained by the expert panel were assessed through focus groups with children (*n* 27, *M age* = 10·2, 15 girls). Children aged 8–12 years were recruited at primary schools. Parents signed consent for their child. Focus groups consisted of 5–8 children and were held at the primary schools. Following a brief introduction, children were asked to complete the questionnaire on paper and raise their hand if they believed a question, explanation or answering option was unclear, poorly worded or confusing. They were encouraged to elaborate on their feedback to the researcher (IvL), who noted all comments. Children received a small token (i.e. a coloured pencil set) for their participation.

### Phase 2: Reliability and validity testing

#### Reliability testing

After final adjustments were made in response to the pilot test results, the questionnaire was administered to 608 children (*M* age = 9·7, 38 % girls). As a rule of thumb, we aimed to include ten children for each item in the questionnaire, resulting in a required minimum sample size of 540 children^(45)^. Recruitment was done via primary schools and a sports club. Efforts were made to include schools from neighbourhoods with varying socio-economic positions to ensure a diverse sample. A total of thirteen primary schools and two sports clubs in the south of the Netherlands were approached, of which five schools and one sports club agreed to participate. The schools and sports club were visited between June 2022 and September 2023, and the questionnaires were administered in class, either on paper or digitally, depending on whether the children had access to a computer.

The responses (scored as 1 = ‘correct’ or 0 = ‘incorrect’) to the 608 completed questionnaires were first scrutinised by considering the difficulty index *P* of each item. This index is calculated as *P = R/T*, where *R* is the number of correct responses and *T* is the total number of responses. Further, we calculated an item discrimination index (DI) using the formula *DI = (U – L)/n*. First, the total number of students in the upper 27 % (those with the highest overall test scores) and the lower 27 % (those with the lowest scores) was determined. Next, for each item, *DI* was calculated, where *U* represented the number of children in the upper 27 % who answered the item correctly, *L* was the number of children in the lower 27 % who answered correctly and *n* was the number of children in the largest of these two groups^(46)^. A higher *DI* indicates better item discrimination between children with (presumably) higher food literacy and those with lower food literacy. For further analysis, we omitted all items considered as either too easy (*P* > 0·85) or too difficult (*P* < 0·15). We further removed those items that had a *DI* < 0·20 (based on the methods by Quaigrain and Ahrin)^(46)^.

#### Construct validity

We examined the multidimensionality of the questionnaire with explorative factor analysis. Further, to gain some insight into the convergent and divergent validity of the scale, all children were asked to indicate how much they agree with the statements ‘I enjoy baking and cooking’ and ‘I don’t like food’ on separate nine-point rating scales. We hypothesised that the first statement would positively correlate with the total score on the DCFLQ, as children who naturally like being involved in food preparation might also be more food literate, whereas the latter statement would negatively correlate with food literacy. We computed Pearson’s correlation analyses between the mean rating of each statement and the total score on the final DCFLQ.

Likewise, we asked children how much they agreed with two statements from the HLS-Child-Q15^(47)^, a questionnaire measuring health literacy, using a four-point scale. We chose one statement that had overlap with food (‘It is difficult for me to find out whether a food is healthy’) and a question unrelated to food (‘I find it hard to adhere to traffic rules and regulations’). For the first statement, we hypothesised that it would positively correlate with food literacy. For the latter statement, we expected to find no meaningful correlation with food literacy. Considering the ordinal scale with limited response options for these ratings, we calculated Spearman’s rank-order correlation coefficients.

We supplemented the above correlation analyses by also examining whether age is correlated with food literacy, arguing that with increasing age children become more knowledgeable of the world in general including food literacy. In other words, we expected a positive correlation between age (in years) and the total DCFLQ score.

Note that while we intended to test divergent validity with a subsample of children whom we expected to score low on food literacy (as preregistered), it was not feasible to obtain the required sample size of 30 (Cohen’s *d* = 0·65, alpha rejection criterion of 5 %, power of 80 %).

#### Test–retest analysis and internal consistency

Of the 608 children, 357 (*M* age = 9·7, 48 % girls) completed the questionnaire twice to assess test–retest reliability. The second completion took place 12–16 d after the initial one and followed the same procedure as the first questionnaire completion. Test–retest reliability was evaluated with intra-class correlation (ICC), with ICC above 0·75 being marked as ‘good’^(48)^.

Cronbach’s *α* was used to assess the internal consistency of the questionnaire. An *α* > 0·70 is considered an acceptable threshold^(45)^, although an *α* > 0·80 is considered an indicator of good internal consistency.

## Results

### Phase 1

#### Expert panel

The initial item pool consisted of 189 items, which were subsequently presented to and critically evaluated by a subgroup of experts from the panel (*n* 7). The experts scored each item as ‘irrelevant’, ‘relevant’ or ‘amendments are necessary’. In the latter case, experts were asked to provide feedback. Additionally, experts were encouraged to provide feedback on the general structure of the questionnaire, including its layout, the grouping of questions and the language used.

Based on the input, items were revised or removed from the questionnaire. Items were removed if two or more experts found them irrelevant. The revised list, consisting of 134 items, was then again sent to the expert panel (*n =* 11). To condense the set of questions, experts were asked to select the thirty items they found most relevant for inclusion in the questionnaire. Questions that were marked as relevant by three or more experts remained in the questionnaire (sixty-five items).

After the second round of adjustment, a panel meeting with a subgroup of the experts (*n* 6, including a psychologist, a paediatrician, a pedagogue and domain-specific experts) was organised to discuss the questions and questionnaire structure based on the feedback that had been given previously. This resulted in slight changes to the questionnaire (i.e. the removal and addition of some items), leading to a seventy-two-item questionnaire.

#### Pilot testing with children

Based on children’s feedback, the wording of several questions was refined. In addition, children agreed that the final questionnaire should not exceed thirty-five questions. In response to this feedback, a selection was made by two experts (a pedagogue and a domain-specific expert) and the researcher (IvL), aiming to retain at least one question per domain. As a result, questions were removed from domains that contained multiple items, leading to a final questionnaire comprising thirty-four questions. Some of these questions included multiple items (e.g. the single question *‘indicate for each of the following products whether salt is added: tomatoes; olive oil; canned soup; cheese’* comprised four items), contributing to a total of fifty-four items in the final questionnaire.

### Phase 2

#### Reliability testing

The difficulty index *P* was determined for each of the fifty-four questionnaire items, identifying eleven items as either too easy (*P* > 0·85, 9 items) or too difficult (*P* < 0·15, 2 items). Further, items with a discrimination index *DI* below the threshold of *DI* ≥ 0·20 were removed, resulting in the additional elimination of one item. This process left a final set of forty-two items. Because some items were combined within a single question, the final questionnaire comprised twenty-nine questions (see online supplementary material, Supplemental 1 for the final questionnaire).

#### Construct validity

Although we found that the scale was suitable for factor analysis (Kaiser-Meyer-Olkin test (KMO) = 0·76, Bartlett’s test: χ^2^ = 4631·19, *P* < 0·001) and parallel analysis suggested exploring 2–5 factor solutions, none of the multifactorial solutions obtained from the factor analysis proved to be satisfactory despite careful consideration. The factors either included too few items with acceptable loadings (less than three items), lacked meaningful interpretability or the identified dimensions showed unacceptable internal consistency (i.e. Cronbach’s *α* < 0·60). Consequently, the questionnaire was treated as a unidimensional instrument.

We found a small but significant positive correlation between DCFLQ score and ratings for the statement ‘I like baking and cooking’, *r* = 0·11, *P* < 0·001, 95 % CI: 0·03, 0·19. There was a small but significant negative correlation between DCFLQ score and agreement with the statement ‘I don’t like food’, *r* = –0·08, *P* = 0·05, 95 % CI: –0·16, 0·00. Likewise, for ratings on health literacy (‘It is difficult for me to find out whether foods are healthy’ and ‘I find it hard to adhere to traffic rules and regulations’), we found small but significant negative correlations with total DCFLQ score, Spearman’s *rho* = –0·13 (*P* < 0·01, 95 % CI: –0·21, –0·05) and –0·16 (*P* < 0·01, 95 % CI: –0·24, –0·08), respectively (see Table 2). Finally, we found a small but significant positive correlation between age and DCFLQ score, *r* = 0·21, *P* < 0·001, 95 % CI: 0·14, 0·29 (see a breakdown by age group in Table 3).

**Table 2.** Convergent and divergent validity questions and their correlation to the DCFLQ

	Spearman’s *rho*	*P*	95 % CI
It is difficult for me to find out whether foods are healthy	–0·13	< 0·01	–0·21, –0·05
I find it hard to adhere to traffic rules and regulations	–0·16	< 0·01	–0·24, –0·08
	**Pearson’s *r***	***P***	**95 % CI**
I like baking and cooking	0·11	< 0·01	0·03, 0·19
I do not like food	–0·08	0·05	–0·16, 0·00

DCFLQ, Dutch Children’s Food Literacy Questionnaire.

**Table 3.** Average scores on the DCFLQ per age group

Age	Number of children (*n*)	Score on DCFLQ	
		Mean	sd
8	87	18·9	6·64
9	195	20·5	6·20
10	178	22·2	6·19
11	117	23·0	6·10
12	31	23·7	7·23

DCFLQ, Dutch Children’s Food Literacy Questionnaire. Minimum score to be obtained: 0; maximum score to be obtained: 42.

#### Test–retest analysis and internal consistency

Considering the results from the subset of children who filled out the questionnaire twice, we found that the test–retest reliability of the DCFLQ was good, ICC = 0·81, *P* < 0·001, 95 % CI: 0·76, 0·85.

Finally, the internal consistency of the DCFLQ was determined, showing a Cronbach’s *α* = 0·80, indicating good internal consistency.

## Discussion

The aim of the current study was the development and initial validation of the DCFLQ. The DCFLQ consists of twenty-nine questions (forty-two items) and demonstrated good internal consistency and test–retest reliability, indicating its potential as an effective tool for assessing food literacy among 8- to 12-year-old Dutch children. The scale is presented as a unidimensional instrument, similar to that of Stjernqvist and colleagues^(33)^, who also identified a single hierarchical dimension reflecting overall ‘child food literacy’ with good internal consistency. It should be noted that Stjernqvist and colleagues identified five subdimensions; however, the majority of these subscales had Cronbach’s *α* values below the threshold of acceptability^(33)^, which is similar to our outcomes. Like the approach taken by Dallant and colleagues^(49)^, we developed our questionnaire based on existing instruments from other countries. Rather than including sustainability as a distinct subdimension, we integrated it throughout the other questions. In terms of the number of items, our questionnaire is similar to existing instruments, which range from 25^(49)^ to 46 questions^(39)^. However, while the brevity of the questionnaire (twenty-nine questions) is advantageous for children, it limits its depth. Amin and colleagues also highlighted this concern, noting that the use of brief questionnaires for evaluating nutrition education programmes should be carefully considered^(15)^.

The total score on the DCFLQ correlated weakly but meaningfully with liking of food. Furthermore, as one would expect, food literacy positively correlated with age. These correlations provide initial empirical support for the validity of the DCFLQ.

The present study also reveals limitations. First, although the current questionnaire aims to assess food literacy, the majority of retained items primarily target the *knowledge* component. Future questionnaires could place greater emphasis on skill-related items, as food literacy also encompasses the ability to *apply* food knowledge. Including more items that assess food-related skills could improve the validity and applicability of the questionnaire. In addition, for some of the items that assess attitudes or applied knowledge, a Likert-type response format may be more appropriate than the current ‘yes’, ‘no’ or ‘don’t know’ options. Future studies could explore whether these items should be reformulated and whether an alternative response scale would improve the measurement properties of the questionnaire. One other recommendation is to develop a complementary skills checklist for teachers and other professionals, enabling them to assess food-related skills (e.g. meal preparation and food handling) during hands-on activities like cooking workshops. In addition, efforts to establish convergent and divergent validity through comparisons with validated scales measuring constructs that are either related or unrelated to food literacy would strengthen the DCFLQ.

The majority of children participating in the validation phase were boys (62 %), primarily due to the inclusion of a sports club with a predominantly male membership. However, analysis revealed no significant differences between boys and girls on the main outcome measures, suggesting that this gender imbalance did not bias the results. We did not assess socio-economic background in the questionnaire, which is a suggestion for future research. However, we aimed to include a diverse sample of children from various socio-economic backgrounds by recruiting children from different neighbourhoods and regions in the south of the Netherlands.

One might question the need for another child food literacy scale, given the existence of several validated instruments. However, food literacy instruments are not easily amenable to direct translation. As noted by Doustmohammadian and colleagues^(39)^, cultural norms, traditions and social practices surrounding food consumption vary widely across different countries and regions, shaping individuals’ knowledge, skills and behaviours related to food. This was further corroborated by Sproesser and colleagues^(50)^, who observed cultural variation in eating habits, including differences in meal composition, portion sizes and attitudes to food. For example, in the Netherlands, children typically eat a cold lunch (mostly home-made sandwiches) rather than a warm meal provided by the school, which is in contrast to most other countries. As a result, questions related to school meals may lack relevance for Dutch children. Simultaneously, while the DCFLQ is appropriate for use in the Netherlands, its relevance may be limited in other countries. Food literacy is inherently contextualised.

While tailoring instruments to cultural contexts enhances their relevance in such context, it limits their utility for cross-cultural comparisons. Therefore, we emphasise the importance of international efforts to attain consensus not only on the definition of food literacy^(17,19)^ but also on the methodological approaches to developing future instruments. Such consensus could help balance the need for cultural relevance with the ability to conduct meaningful cross-cultural research.

To conclude, the DCFLQ can be used in longitudinal, prospective cohort studies to monitor the developmental trajectory of food literacy and its impact on dietary behaviours and health outcomes among Dutch children. Practically, the DCFLQ can inform educational interventions and policy-making by identifying areas where children may lack knowledge or skills, thereby guiding curriculum development and public health strategies. Additionally, exploring the relationship between food literacy and other factors such as socio-economic position and access to food resources can provide a deeper understanding of how to effectively promote food literacy. Future studies could also focus on the development of food literacy among younger children. Investigating the long-term effects of food literacy on dietary choices and health outcomes would further help to gauge the validity of this instrument.

## Supporting information

van Lier et al. supplementary material van Lier et al. supplementary material

## Supplementary material

For supplementary material accompanying this paper visit https://doi.org/10.1017/S1368980025101651
