# Supplementary material for: The initial development and validation of a child-oriented food literacy questionnaire
Source: Public Health Nutr. 2025 Dec 29;29(1):e26. doi: 10.1017/S1368980025101651 (PMC12895494; doi:10.1017/S1368980025101651)
Supplement: van Lier et al. supplementary material [file S1368980025101651sup001.docx]

**Supplementary data.** Questions included in the Dutch Children’s Food Literacy Questionnaire (DCFLQ).

The questions can be answered with ‘true’, ‘not true’, or ‘don’t know’. The original questionnaire is in Dutch.

| **Item** | **Question** | **Subdomain (see Table 1)** |
| --- | --- | --- |
| 1 | Organic food must comply to many rules. These rules help to protect the environment and animals as much as possible. | Origin |
| 2 | A mushroom is an edible fungus. | Source |
| 3  4 | Some products are of animal origin. Indicate for the following combinations whether the product comes from this animal:   - Ham 🡪 cow. - Honey 🡪 wasp. | Source |
| 5  6  7  8 | Fruit and vegetables grow in different ways. Indicate whether the following statements are true or false:   - Tomatoes grow underground. - Peanuts grow underground. - Mushrooms grow in the dark. - Blueberries grow on trees. | Growing process |
| 9 | A strawberry sold in winter is grown in a greenhouse. | Processing |
| 10 | Most Dutch apples are harvested in September and October. | Processing |
| 11 | In summer, 500g of blueberries costs €3. In winter, 500g of blueberries costs less than €3. | Self-efficacy |
| 12 | Products on the lowest supermarket shelf are more expensive than those found on higher shelves. | Self-efficacy |
| 13  14  15 | Some foods naturally contain sugar, while others have added sugar. Indicate for each of the following products whether sugar has been added:   - Banana - Milk chocolate - Ketchup | Ingredient |
| 16  17  18  19 | Indicate for each of the following products whether it contains a lot of saturated fat:   - Salmon - Crackers - Chocolate - Walnuts | Ingredient |
| 20  21  22  23  24 | Indicate for each of the following products whether salt has been added:   - Potatoes - Olive oil - Tomatoes - Canned soup - Gouda cheese | Ingredient |
| 25 | [includes image of a red cabbage] This picture shows a red cabbage. | Diet composition |
| 26 | [includes image of a pomegranate] This picture shows a pomegranate. | Diet composition |
| 27 | [includes image of cashew nuts] This picture shows cashew nuts. | Diet composition |
| 28 | A handful of unsalted nuts is a healthy snack. | Nutrition and health |
| 29 | Dietary fibre helps your intestines function properly. | Nutrition and health |
| 30 | Liam is allergic to soy. He wonders if he can eat a cookie. On the food label, Liam can find whether the cookie contains soy. | Food labels and information |
| 31 | [includes image of a food label] Some ingredients on the food label are printed in bold. This means that large amounts of these ingredients are present in the product. | Food labels and information |
| 32 | [includes image of a food label] The ingredient listed first is present in the largest quantity. | Food labels and information |
| 33 | Not eating meat one day a week is better for the environment than eating meat every day. | Diet composition |
| 34 | [includes image of a small banana and two kiwis] The picture below shows a small banana and two kiwis. If you eat these in one day, you have eaten enough fruit for that day. | Nutrition and health |
| 35 | Stir-frying is healthier than deep-frying | Nutrition and health |
| 36 | [includes one image of vegetables steamed above boiling water (A) and one image of vegetables boiled in water (B)] The picture below shows two ways of preparing vegetables. If you want to keep as many vitamins as possible, it is better to prepare vegetables as shown in picture A than in picture B. | Cooking skills |
| 37 | Milk that has gone bad smells sour. | Food safety |
| 38 | If you use a cutting board to cut raw meat, you should not use the same board immediately afterwards to cut cooked meat. | Food safety |
| 39 | It is better to eat your meal while sitting at the table than in front of the TV. | Social norms |
| 40 | Halal meat is meat that comes from abroad. | Culture and traditions |
| 41 | If a product shows the abbreviation “TGT” (“use by”) on the package, it means that you can still eat the product even if the date mentioned was yesterday. | Reducing food waste |
| 42 | When you cut an apple in half, it turns brown. You can prevent this by sprinkling lemon juice over the apple. | Recycling foods |
